# Correction to “Gliovascular alterations in sporadic and familial Alzheimer's disease: *APOE3* Christchurch homozygote glioprotection”

**DOI:** 10.1111/bpa.70138

**Published:** 2026-08-25

Henao‐Restrepo J , López‐Murillo C , Valderrama‐Carmona P , Orozco Santa N , Gomez J , Gutiérrez‐Vargas J , et al. Gliovascular alterations in sporadic and familial Alzheimer's disease: *APOE3* Christchurch homozygote glioprotection. Brain Pathol. 2023; 33(2):e13119. 10.1111/bpa.13119 36130084 PMC10041169

1. The description of the human samples did not fully describe the criteria used to select cases included in each experimental analysis. In the Materials and Methods section (“2.1 Human samples”), the following sentence should be added after “…comorbidities (Table 1 and Table S1)”:

“Postmortem cases included in each experiment were selected according to tissue availability and suitability for the specific analyses performed. A proportion of the postmortem cases overlapped with those included in our previous publications [1, 2], reflecting the use of the same finite brain bank collection and the selection of tissue according to its availability and suitability for the different analyses performed.”

2. The methodological description did not include specific information regarding the processing of the *APOE3* Christchurch tissue. In the Materials and Methods section (“2.2 Immunohistochemistry and immunofluorescence”), the following paragraph should be added before the sentence, “Samples were fixed and cut to 50 μm thick for Nissl staining and immunolabeling”:

“Prior to sectioning, postmortem brain tissue underwent fixation according to the routine procedures of the University of Antioquia Brain Bank. Pre‐analytical handling varied depending on tissue availability and prior processing history. Some tissue fragments underwent a period of formalin fixation before transfer to 4% paraformaldehyde for 72 h, with replacement of the fixative every 24 h, whereas others were fixed directly in 4% paraformaldehyde for the same period prior to immunohistochemistry and immunofluorescence. The *APOE3* Christchurch brain tissue was processed according to the latter workflow and became available during the final immunostaining series conducted for this study. This pre‐analytical difference was specific to the immunostaining experiments performed during the final staining series and did not apply to the AT8, Aβ, Aquaporin‐4 immunohistochemistry, or Aquaporin‐4/lectin/GFAP immunofluorescence experiments, in which the *APOE3* Christchurch case was processed together with the remaining postmortem cases. All tissue sections included in the corresponding quantitative analyses were subsequently processed, stained, acquired, and analyzed using the same protocols, imaging settings, and predefined quantitative criteria.”

3. The legends to Figure 8A and A′ did not disclose that representative Claudin‐5 immunohistochemical images had been previously published in a related article from our laboratory. Figure 8A and A′ should be replaced with alternative representative images from the corresponding original datasets as follows:

**FIGURE 8:**
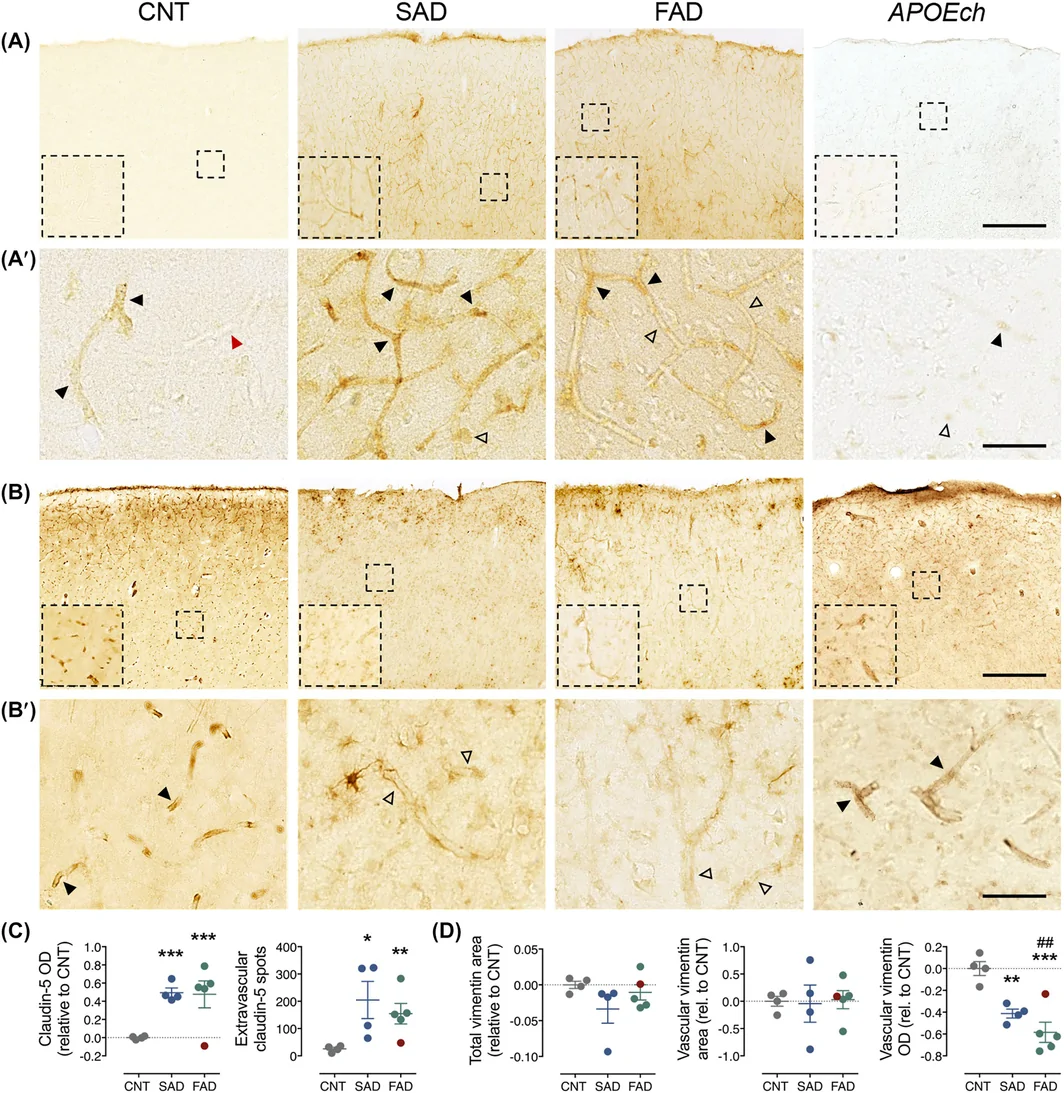
(A) Replacement representative images for the SAD and FAD groups, including the corresponding higher‐magnification insets. (A′) Replacement representative images for the Control, SAD, and FAD groups.

We apologize for these errors.
